# The oxidative and thermal stability of optimal synergistic mixture of sesame and grapeseed oils as affected by frying process

**DOI:** 10.1002/fsn3.2774

**Published:** 2022-02-15

**Authors:** Maryam Khakbaz Heshmati, Maryam Jafarzadeh‐Moghaddam, Akram Pezeshki, Rezvan Shaddel

**Affiliations:** ^1^ 56947 Department of Food Science and Technology Faculty of Agriculture University of Tabriz Tabriz Iran; ^2^ 185149 Department of Food Science and Technology Faculty of Agriculture and Natural Resources University of Mohaghegh Ardabili Ardabil Iran

**Keywords:** grapeseed oil, oxidative stability, sesame oil, thermal stability

## Abstract

In spite of grapeseed oil high contents of linoleic acid, its oxidative stability is relatively low, and mixing with more stable oils such as sesame oil can be a good way to improve the oxidative stability of this oil. The aim of this study was to increase the oil oxidative stability by producing an optimum formulation due to the combination of grapeseed and sesame oil. For this purpose, some of the qualitative properties of the optimum formulation were investigated during frying process. For finding the best formulation, the quantities of 0%, 25%, 50%, 75%, and 100% of sesame oil were blended with 100, 75%, 50%, 25%, and 0% of grapeseed oil. The results show that the highest percentage of fatty acid in various samples (sesame oil, grapeseed oil, and mixed formulations) is related to the linoleic acid, followed by oleic, palmitic, and stearic acid. In conclusion the addition of sesame oil to grapeseed oil increased the number of phenolic compounds, antioxidant strength, and oxidative stability of the mixed oil samples. Considering the price of the product and the importance of the nutritional quality and stability of the oil, combining 75% sesame oil and 25% grapeseed oil has the best nutritional quality and lower cost than pure sesame oil formula. After frying process, comparison of sesame and grapeseed oil different factors with national Iranian standard limits showed that the parameters of acid number and peroxide value were more than Iran’s national standard, but the content of polar compounds was within the permissible content. Finally, the mixture of sesame and grapeseed oil is not suitable for long‐term heating and frying.

## Graphical abstract

Key findingsa) Mixing grapeseed oil with sesame oil can be a good way to improve the oxidative stability of grapeseed oil.b) The mixture of 75% sesame oil and 25% grapeseed oil as the optimum formulation had the best nutritional quality and lower cost.c) The optimum mixture of sesame oil and grapeseed oil is suitable to use as a cooking or salad oil and not for long‐term frying.

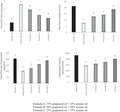

## 1 INTRODUCTION

Frying is one of the most common and widely used food processing methods in the world. Fried products are very popular among consumers due to their crispy texture and pleasant flavor. Accordingly, the enormous economic value of the fried foods trade is estimated to be about 83 billion dollar per year in the United States (Choe & Min, 2007). The grapeseed oil contains a high amount of linoleic acid (68%–76%) which is the essential fatty acid of the human body. The use of linoleic acid is necessary for the human diet in terms of the tissues’ functionality and maintenance of the body (Negro et al., 2003). However, these unsaturated fatty acids are more susceptible to some adverse reactions (Board, 2013). There are several types of tocopherols and tocotrienols in grapeseed oil such as α, β, ϒ, and δ in which the ϒ‐tocotrienols are the most important ones, according to Kim et al. (2008). The amount of α‐tocopherol in grapeseed oil is also high. It was indicated by some researchers (e.g., Shahidi and Zhong (2020)) that the presence of a wide range of natural antioxidants in vegetable oils can protect the oil against thermal degradation, transfer their health benefits and reduce the incidence of various cancers and oxidation stresses. However, during the heat treatment of vegetable oils, their physical and chemical properties are subjected to some deteriorations such as increasing the acid number and peroxide value, reducing the oxidative stability, changing the type or amount of pigments, and eventually forming dimers and polymers that increase the viscosity of the oil (Casal et al., 2010).

Grape seeds can be obtained from the waste of the juicer factories. Therefore, in terms of raw material costs, grapeseed oil is the economical raw material (Negro et al., 2003). On the other hand, since the combination of sesame oil with some edible oils improves the nutritional value and stability of the mixture oil during frying and cooking process, so the combination of oils is the most cost‐effective process for oil modification (Casal et al., 2010).

The effects of frying process have been tested by different researchers on the chemical composition of various vegetable oils (such as olive oil and sunflower oil) (Romano et al., 2012; Normand et al., 2006). However, there are few researches on the effect of frying process on the quality characteristics of the oil mixtures (Khakbaz Heshmati et al., 2019; Khakbaz Heshmati et al., 2021). In this study, the mixtures of sesame oil and grapeseed oil were prepared in different proportions and their physicochemical properties, fatty acid profile, and antioxidant activity were examined. After finding an optimum formulation, some of the qualitative properties of oil were investigated during frying process. This study aimed to evaluate the effects of frying process on the oil optimum formulation for evaluating the resistance of this formulation to the thermal and oxidative damages as a nutritious and free of synthetic antioxidant oil that is suitable for frying.

## 2 MATERIALS AND METHODS

### 2.1 Preparation of sesame oil and grapeseed oil formulations

The chemical materials used in this study include chloroform, ethanol, sodium hydroxide, acetic acid, potassium iodide, sodium thiosulfate, hexane, isopropanol, p‐Anisidine, toluene, petroleum ether (Merck, Germany). The virgin sesame oil (extracted by the cold press method) was purchased from Saman sesame oil factory, Tehran, Iran. Grape seeds type of *Vitis Vinifera L*. (Var. *Siahe*) isolated from the grape pulp (the byproduct of the grape juice factory, Urmia, Iran) and grapeseed oil extracted from the grape seeds in the laboratory. For extraction of grapeseed oil, the seeds dried (up to 6% moisture content) at 50°C for 2 h and milled. The grapeseed oil was extracted by Soxhlet method using petroleum ether solvent at 90°C for 6 h. The extraction of sesame and grapeseed oil both was done simultaneously in one day. To prepare five different formulations (100% sesame oil, 100% grapeseed oil, Formula A: 25% sesame oil and 75% grapeseed oil, Formula B: 50% sesame oil and 50% grapeseed oil, Formula C: 75% sesame oil and 25% grapeseed oil), sesame oil and grapeseed oil were weighed (w/w) and poured in a beaker and completely homogenized by a magnetic stirrer. The prepared formulations were evaluated for peroxide value, iodine value, fatty acid composition, oxidative stability, total phenol, and antioxidant activity. All tests were done within 1 week after extraction of the sesame and grapeseed oil.

#### 2.1.1 Quantitative and qualitative experiments of oil the formulations

##### Composition of fatty acids

To determine the composition of fatty acids, the methyl ester of fatty acids was prepared according to AOCS Surplus Method (AOCS, 2009, Ce 1k‐09). Then, a Techcomp GC7900 gas chromatography machine equipped with a flame ionization detector was used (China). The capillary column was 60‐meter‐long, which had a diameter of 0.25 mm and a thickness of 0.32 μm. The oven temperature was adjusted to 198°C and the injector and detector temperatures were adjusted to 250°C.

##### Peroxide value

The peroxide value was measured using AOCS Surplus method (AOCS, 2003, Cd 8‐53). At first, 30 ml of acetic acid‐chloroform (3:2) solution with 0.5 ml of saturated solution of potassium iodide was added to 5 g of oil samples and kept in the darkroom. After 1 min, 30 ml of distilled water with 0.5 ml of starch indicator (1% w/v) was added to the oil samples to appear blue color in the presence of peroxide. Then, the titration was performed with 0.01N sodium thiosulfate until the blue color disappeared.

##### Iodine value

Iodine values were determined by AOCS Surplus method (AOCS, 1993, Cd 1‐25).

##### Oxidative stability

The oxidation stability of samples was determined by the RANCIMAT method EN 14112 using a RANCIMAT instrument (model 893; Professional Biodiesel RANCIMAT; Metrohm AG, Herisau, Switzerland). For this purpose, 3 g of the oil samples was tested at 120°C and the aeration rate of 20 liters per hour (Farhoosh & Moosavi, 2007).

##### Total phenolic compounds

Total phenol was measured according to the Karimkhani et al. (2016) method. The measurement of phenolic compounds was performed based on the Folin–Ciocalteu colorimetric method.

##### Antioxidant activity

Total antioxidant activity was measured according to the Velioglu et al. (1998) method. The samples absorbance was measured at 593 nm by Shimadzu UV‐1601 Spectrophotometer, Japan.

### 2.2 Preparation of the oil optimum formulation

Based on the results of physicochemical and antioxidant properties evaluation as well as the product price of the grapeseed oil and sesame oil combination, the optimal mix of sesame oil and grapeseed oil was selected (Khakbaz Heshmati et al., 2019). For this purpose, 75% w/w of sesame oil and 25% w/w grapeseed oil was mixed and completely homogenized at ambient temperature.

#### 2.2.1 Evaluation of the oil optimum formulation quality during frying process

To evaluate the oil quality during frying process, the oil optimum formulation was prepared without any additives and kept at a temperature of ‐18°C. Also, a commercial oil (with the formulation of sunflower oil, soybean oil, palm olein oil, palm super olein oil, and canola oil) which had purchased immediately after production was weighted in a glass container and kept at a temperature of ‐18°C. All samples were heated in the oven with a temperature of 180°C and at times of 0 (control sample), 30, 60, and 90 min, then were cooled in the desiccator and were stored at ‐18°C until the test time. The acid value (Pardo et al., 2009), peroxide value (AOCS, 2003, Cd 8‐53), total phenol (Velioglu et al. 1998) compounds of optimum formulation were evaluated; also, total polar compounds and p‐Anisidine value were evaluated according to the following methods.

##### Measurement of p‐Anisidine value

p‐Anisidine value was determined by the AOCS Official method (AOCS, 2017, Cd 18‐90) using 0.2–0.4g samples, glacial acetic acid, and analytical grade isooctane, p‐Anisidine (99%) (ALDR‐A88255, Aldrich), and a quartz cuvette. A blank was prepared for each sample and the absorbance of the samples was read in the visible and ultraviolet ranges of the UV/VIS Lambda 20 Spectrophotometer (Perkin Elmer, Germany). The p‐Anisidine value was calculated using equation 1:

(1) Anisidinevalue=(25×(1.2AS‐AB))/W

where AS is the absorption of the solution after reaction, AB represents absorption before reaction and M denotes mass of sample in grams.

##### Total polar compounds

The measurement of total polar compounds was done using a Knauer HPLC made in Germany (Schulte, 2004).

### 2.3 Statistical analysis

All tests were performed in three replications, and statistical analysis was done in a completely randomized design using SPSS software (Version 22, USA). Duncan's multiple range test was employed to determine any significant difference between mean values of different characteristics and a 95% significance level (*p* ≤ .05) was adopted.

## 3 RESULTS AND DISCUSSION

### 3.1 Sesame oil and grapeseed oil formulations

#### 3.1.1 Quantitative and qualitative experiments of the oil formulations

##### Composition of fatty acids

The fatty acid composition of sesame oil, grapeseed oil, and A, B, and C formula has been presented in Table 1. According to the data in the table, linoleic acid, oleic acid, palmitic acid, and stearic acid, respectively, compose the highest percentage of fatty acid in all oil formulations (Sesame oil, grapeseed oil, and A, B, and C formula). Comparison of sesame oil and grapeseed oil fatty acid profile shows oleic acid content of the sesame oil is significantly higher than grapeseed oil, while linoleic acid of the grapeseed oil is much more than sesame oil (Table 1). On the other hand, the amount of saturated fatty acids in sesame oil is significantly (*p* ≤ .05) higher than grapeseed oil. This result is due to the presence of a higher amount of palmitic acid and the higher oleic acid content of sesame oil. The amount of monounsaturated fatty acids of sesame oil was significantly (*p* ≤ .05) higher than grapeseed oil due to the higher content of oleic acid. According to Table 1, by increasing the proportion of sesame oil (A, B, and C formula) linoleic and stearic acid content decreases while the amount of oleic acid and palmitic acid increases. Also increasing the proportion of sesame oil causes decreasing in the mixed oils polyene index. However, this reduction does not result in losing the quality of the mixed oil under the acceptable level of WHO (World Health Organization) or FDA (Food and Drug Administration).

**TABLE 1:** The initial fatty acid composition (%) of sesame oil and grapeseed oil and the mixing formulas

Fatty acid composition	Sesame oil	Grapeseed oil	Formulated Oil		
			A	B	C
Meristic acid (14: 0C)	0.05 ± 0.00^b^	0.05 ± 0.00^b^	0.07 ± 0.01^ab^	0.06 ± 0.01^b^	0.06 ± 0.01^b^
Palmitic acid (16: 0C)	11.59 ± 0.06^a^	8.64 ± 0.05^d^	8.81 ± 0.04^d^	9.45 ± 0.14	10.52 ± 0.19^b^
Palmitoleic acid (16: 1C)	0.16 ± 0.01^b^	0.32 ± 0.03^a^	0.30 ± 0.02^a^	0.29 ± 0.01^a^	0.18 ± 0.02^b^
Margaric acid (17: 0C)	0.04 ± 0.00^b^	0.11 ± 0.02^a^	0.10 ± 0.02^a^	0.05 ± 0.01^a^	0.06 ± 0.02^b^
Stearic acid (18: 0C)	2.66 ± 0.01^c^	4.08 ± 0.04^a^	3.82 ± 0.09^a^	3.18 ± 0.14^b^	3.12 ± 0.09^b^
Oleic acid (18: 1C)	40.23 ± 0.16^a^	21.88 ± 0.26^e^	26.94 ± 0.21^d^	30.21 ± 0.26^c^	34.57 ± 0.15^b^
Linoleic acid (18: 2C)	42.52 ± 0.17^e^	62.50 ± 0.28^a^	57.71 ± 0.33^b^	51.61 ± 0.36^c^	48.58 ± 0.25^d^
Linolenic acid (18: 3C)	1.27 ± 0.04^a^	0.88 ± 0.06^c^	0.93 ± 0.04^b^	1.18 ± 0.04^b^	1.27 ± 0.03^a^
Arachidic acid (20: 0C)	0.51 ± 0.01^a^	0.23 ± 0.02^c^	0.21 ± 0.02^c^	0.47 ± 0.04^b^	0.54 ± 0.01^a^
Eicosanoic Acid (20: 1C)	0.24 ± 0.02^b^	0.21 ± 0.03^bc^	0.20 ± 0.01^c^	0.28 ± 0.02^a^	0.25 ± 0.03^ab^
Beanic acid (22: 0C)	0.21 ± 0.01^d^	0.88 ± 0.07^a^	0.61 ± 0.03^b^	0.45 ± 0.07^c^	0.40 ± 0.01^c^
Lignoceric acid (24: 0C)	0.23 ± 0.01^a^	0.05 ± 0.01^c^	0.10 ± 0.01^b^	0.11 ± 0.01^b^	0.15 ± 0.01^b^
SFA	15.29 ± 0.15^a^	14.07 ± 0.21^c^	13.72 ± 0.21^c^	13.76 ± 0.11^c^	14.84 ± 0.22^b^
MUFA	40.63 ± 0.45^a^	22.41 ± 0.39^e^	27.44 ± 0.38^d^	30.77 ± 0.37^c^	35.00 ± 0.31^b^
PUFA	43.79 ± 0.74^e^	63.38 ± 0.88^a^	58.58 ± 0.71^b^	52.54 ± 0.69^c^	49.76 ± 0.49^d^
PUFA/SFA	2.86 ± 0.11^e^	4.50 ± 0.13^a^	0.07 ± 0.01^ab^	0.06 ± 0.01^b^	0.06 ± 0.01^b^

Note

SFA: Saturated Fatty Acids, MUFA: Monounsaturated Fatty Acids, PUFA: Polyunsaturated Fatty Acids.

Different superscripts represent significant differences at *p* < .05.

Formula A: 75% grapeseed oil + 25% sesame oil.

Formula B: 50% grapeseed oil + 50% sesame oil.

Formula C: 25% grapeseed oil + 75% sesame oil.

##### Peroxide value

**FIGURE 1:**
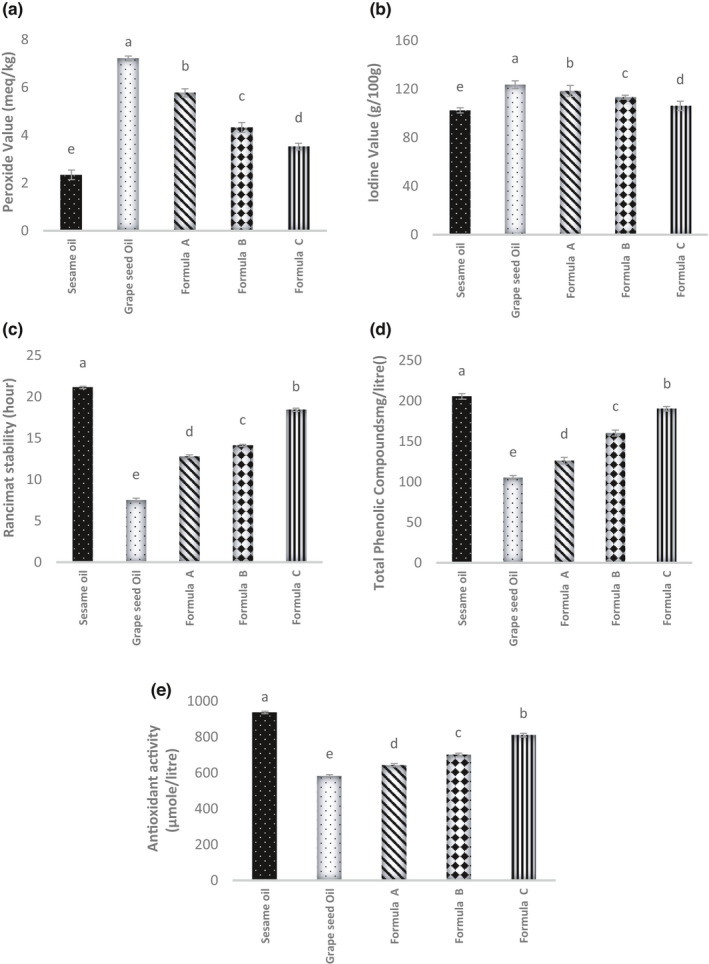
(a‐e) Peroxide value, Iodine value, Rancimat stability, Total phenolic compounds, and Antioxidant activity of sesame and grapeseed oil and formulated oils (Formula A: 75% grapeseed oil + 25% sesame oil, Formula B: 50% grapeseed oil + 50% sesame oil, Formula C: 25% grapeseed oil + 75% sesame oil), respectively; Different superscripts represent significant differences at *p* < .05

Examining the peroxide value as an indicator of the primary oxidation products is a particular method to ensure the oil oxidative stability. Therefore, the less peroxide value, the more oil quality (Gouveia et al., 2017). The results of the sesame, grapeseed oil, and A, B, and C formula peroxide analysis (one week after extraction) are shown in Figure 1a. The highest and the lowest peroxide value are related to the grapeseed oil (7.21 meq kg^−1^) and the sesame oil (2.34 meq kg^−1^), respectively. With the increase in the sesame oil proportion in the oil formulation, the peroxide value significantly decreased (*p* ≤ .05). The peroxide value of the sesame oil, 25% grapeseed oil + 75% sesame oil, and 50% grapeseed oil + 50% sesame oil was acceptable according to the Iranian national standard (the maximum peroxide value for edible and mixed oils is 5 meq kg^−1^). Also, the peroxide value of the mixture of 25% sesame oil and 75% grapeseed oil (5.77 meq kg^−1^) is close to the (5 meq kg^−1^) mentioned in INSO standard No. 9131 (INSO, 2007) and INSO standard No. 5950 (INSO, 2013).

As shown in Figure 1a, mixing 50% and 75% of sesame oil with grapeseed oil reduced the peroxide value to the acceptable level of the national standard of Iran. As a result, the use of sesame oil in these amounts can guarantee the oxidative stability of the mixture oil. On the other hand, the comparison of the peroxide value of this research samples with the Codex Standard for Edible Fats and Oils (CODEX STAN 19‐1981) suggests that the peroxide values of all samples are within the acceptable limits of the codex standard (According to this standard, the maximum peroxide value is 10 meq kg^−1^). The difference of the peroxide values reported for grapeseed oil at different researches (Pardo et al., 2009) with this research can be attributed to the type of grape variety.

##### Iodine value

The oil with a high iodine value contains a large number of double bonds and usually has low oxidative stability (Gharby et al., 2017). The results of the iodine value of sesame oil, grapeseed oil, and their mixtures are shown in Figure 1b. The iodine value of sesame oil and grapeseed oil is 102 g and 128 g, respectively. Youssef et al. (2013), Gharby et al. (2017), and Olasunkanmi et al. (2017) in the same results, reported the iodine value of sesame oil was 103 g, 117 g, and 121.11 g, respectively. Adding sesame oil to the grapeseed oil at 25%, 50%, and 75% ratios reduced the iodine value to 118.41, 113.29, and 106.19 g, respectively (Figure 1b). With the increase in sesame oil ratio, the unsaturated characteristic of grapeseed oil decreased.

##### Oxidative stability

Measuring the oxidative stability of oils (by Rancimat method) allows comparing the degradation degree during heating (Farhoosh et al., 2009; Rajaei et al., 2021). The turning point of the oxidation curve is defined as the induction period and, the length of this point is considered as the oxidative stability of oil (Abdel‐Razek et al., 2011). The result of measuring the oxidative sensitivity of pure oils (sesame and grapeseed oil) and their mixtures is shown in Figure 1c. As shown in Figure 1c, the highest and the lowest stability refer to sesame oil and grapeseed oil, respectively. By increasing the sesame oil ratio, stability increases significantly (*p* ≤ .05). According to INSO No. 5950 (INSO, 2013), the minimum stability value (Rancimat at 110°C) is 12 h. Therefore, the stability of sesame oil (21.12 h) and the stability of all mixtures of grapeseed oil and sesame oil (18.42‐12.79 h) are within the acceptable range of Iranian national standard except grapeseed oil, which its stability (7.53 h) is significantly lower than the standard level. Interestingly, recent studies reported that if the stability of the heated oil exceeds 2.3 h, that oil is still safe and considered acceptable in terms of taste properties (Farhoosh et al., 2009). Based on this report, all of the oils investigated in this study show very good stability. This result demonstrates the potential of sesame oil to improve the stability of grapeseed oil.

##### Total phenolic compounds

Phenols are antioxidant compounds that react with free radicals of lipid and prevent or slow down the initiation of the oxidation process (Hassanien and Abdel‐Razek, 2012). As a result, the presence of these compounds in the oil is important. The results of the total phenol content of different oil samples are presented in Figure 1d. According to this figure, the highest total phenol content observed in sesame oil (205.43 mg l^−1^) and other oils had lower amounts of phenolic compounds. As the ratio of sesame oil increases, the total phenol content increases (*p* ≤ .05). Consistent with the results of this study, Hassanien and Abdel‐Razek (2012) reported that sesame oil contains a high level of phenolic compounds (255 mg l^−1^), and the addition of sesame oil to sunflower and soybean oil increased the total phenolic content and helps preventing the oxidation of these oils.

##### Antioxidant activity

The results of the antioxidant activity of oil samples are shown in Figure 1e , Figure 1e , indicates that the antioxidant activity of oil samples is in agreement with the content of their phenolic compounds. The highest antioxidant activity related to the sesame oil and, this activity increased significantly with increasing sesame oil ratio (*p* ≤ .05). Due to the high natural antioxidant found in the formulated oil (with a certain proportion of sesame oil), the use of such oils in human diet can reduce the adverse effects caused by oxidative stress in the biological systems. The researchers (Hassanien and Abdel‐Razek, 2012) reported that many factors such as fatty acid composition contribute to the rise of oxidative stability caused by the addition of sesame oil but the lignan content plays a more significant role.

### 3.2 Determination of the sesame oil and grapeseed oil optimum formulation quality

The initial quality of the frying oil is one of the factors, which influences the rate of oxidative stability of the oil during frying process. On the other hand, by analysis of freshly extracted oil physical and chemical properties, it will be known what happened to the oilseed in the process of sowing, harvesting, transportation, storage, refining, etc. Table 2 presents some of the initial physical and chemical properties of sesame oil, grapeseed oil, the oil optimum formulation, and the commercial oil. As shown in Table 2, the iodine value of sesame oil and grapeseed oil was significantly higher than commercial oil (*p* ≤ .05). The iodine value is considered a measure of oil unsaturation. The acid value of the oil optimum formulation was also significantly higher than commercial oil. Any physical damages during harvesting, transportation and storage, insect, and fungal contamination, as well as climatic conditions, lead to the activation of oilseed enzymes (Shahidi & Zhong, 2020). Lipolysis of ester bonds in fatty acids increases free fatty acids, mono‐ and diglycerides. One of the main goals of the refining process is removing fatty acids from crude oil since the presence of fatty acids is one of the main limiting factors of oil efficiency in the frying process. The observed differences between the quality of oil optimum formulation and fresh commercial oil may be related to their oxidation stability parameters. According to the results presented in Table 2, the peroxide value, polar compounds, and p‐Anisidine value of the oil optimum formulation were significantly (*p* < .05) higher than commercial oil. The presence of free fatty acids is accrued due to the oil oxidation during harvesting, transportation, storage, and finally, extraction and refining process. Considering the higher content of polyunsaturated fatty acids in oil optimum formulation in comparison with commercial oil (Table 1), the higher peroxide value of the oil optimum formulation was predictable. As shown in Table 2, the peroxide values of sesame oil, grapeseed oil, the optimum formulation of mixed oil, and commercial oil all are within the acceptable (5 milligrams per kilogram) range of Iranian national standard (INSO standard No. 9131 (INSO, 2007) and INSO standard No. 5950 (INSO, 2013)). Comparing the peroxide value of oil samples with Codex standard indicates that the peroxide value of all oil samples is within the acceptable range of Codex standard. According to Codex standard, the maximum permitted peroxide value is 10 mEq kg^−1^ (Popa et al., 2017). The amount of para Anisidine value for the oil optimum formulation is significantly (*p* ≤ .05) lower than commercial oil (Table 2). This result probably is due to the low para Anisidine value of the sesame oil. The oxidative stability parameter indicates the oxidative stability of the oil optimum formulation is more than commercial oil. According to Table 2, the oxidation stability is about 18.42 h for the oil optimum formulation and 17 h for the commercial oil.

**TABLE 2:** Primary physicochemical properties of sesame oil, grapeseed oil, the optimum formulation of sesame and grapeseed oil, and the control sample

Parameters	Sesame oil	Grapeseed oil	Optimum Formula	Commercial oil (Control)
Iodine value (g/100g)	102.43 ± 1.03^b^	123.55 ± 2.4^a^	106.19 ± 1.43^b^	81.32 ± 1.10^c^
Acid value (%)	2.14 ± 0.13^a^	0.26 ± 0.043^d^	0.64 ± 0.02^b^	0.073 ± 0.001^c^
Peroxide value (meq/kg)	2.34 ± 0.11^c^	7.21 ± 0.12^a^	3.52 ± 0.21^b^	1.540 ± 0.10^d^
p‐Anisidine value (Anisidine unit)	1.96 ± 0.03^c^	8.50 ± 0.23^a^	3.25 ± 0.16^b^	7.5 ± 0.85^a^
Polar components (%w/w)	7.00 ± 0.15^a^	4.40 ± 0.03^b^	6.01 ± 0.23^a^	3.21 ± 0.21^b^
Oxidative stability (h)	21.50 ± 0.43^a^	7.53 ± 0.31^c^	18.42 ± 0.70^b^	17 ± 0.50^b^

Note

Each value in the table represents the mean ± standard deviation of triplicate analysis. Different superscripts within the same row represent significant difference at *p* < .05.

The main fatty acid composition of the oil optimum formulation and commercial oil is presented in Table 3. As shown in Table 3, the main fatty acid of the oil optimum formulation was linoleic acid and oleic acid, respectively. The main fatty acid of commercial oil was oleic acid and palmitic acid. The fatty acid profile of oil is a critical factor, which influences the oil oxidative stability during frying. Increasing double bonds in unsaturated and polyunsaturated fatty acids declines the required energy for separating hydrogen from fatty acid. This phenomenon follows by the fatty acid participation in the oxidation, isomerization, polymerization, and cyclization reactions. This results in a decline in the organoleptic quality of frying oil and develops toxic compounds. Besides the abundance of unsaturated fatty acids, the type of unsaturated fatty acids and the rate of being unsaturated are considerable factors in oil oxidative stability. As shown in Table 3, the abundance of polyunsaturated fatty acids in the oil optimum formulation is lower than the commercial oil. Therefore, this fact could be a reason for the high oxidative stability of the oil optimum formulation. The contribution of saturated, monounsaturated, and unsaturated fatty acids of the oil optimum formulation and commercial oil is presented in Table 4. According to Table 4, polyunsaturated fatty acids constitute about 58% of the fatty acids in the oil optimum formulation, whereas they constitute about 25% of the fatty acids in the commercial oil. However, this result is not sufficient alone to determine the higher oxidative stability of the oil optimum formulation and verification of this fact needs the investigation of the physical and chemical properties of the oil during the frying process. Until now, this oil formulation is more suitable for the frying process in comparison to commercial oil.

**TABLE 3:** The main fatty acids composition of the optimum formulation of sesame and grapeseed oil and the commercial oil

Sample	Optimum formula	Commercial oil (Control)
Palmitic acid (16:0)	8.81 ± 0.04	27.54 ± 0.31
Stearic acid (18:0)	3.82 ± 0.09	5.54 ± 0.33
Oleic acid (18:1)	26.94 ± 0.21	42.06 ± 1.19
Linoleic acid (18:2)	57.71 ± 0.33	21.93 ± 1.03
Linolenic acid (18:3)	0.93 ± 0.04	1.57 ± 0.10

**TABLE 4:** The contribution of fatty acids in the optimum formulation of sesame oil and grapeseed oil and commercial oil

Sample	Optimum formula	Commercial oil (Control)
Saturated Fatty Acids	13.98 (%)	32.24 (%)
Unsaturated Fatty Acids	86.02 (%)	67.76 (%)
Monounsaturated Fatty Acids	27.44 (%)	42.12 (%)
Polyunsaturated Fatty Acids	58.58 (%)	25.64 (%)

### 3.3 The qualitative changes in oil optimum formulation during frying

#### 3.3.1 Acid value

**FIGURE 2:**
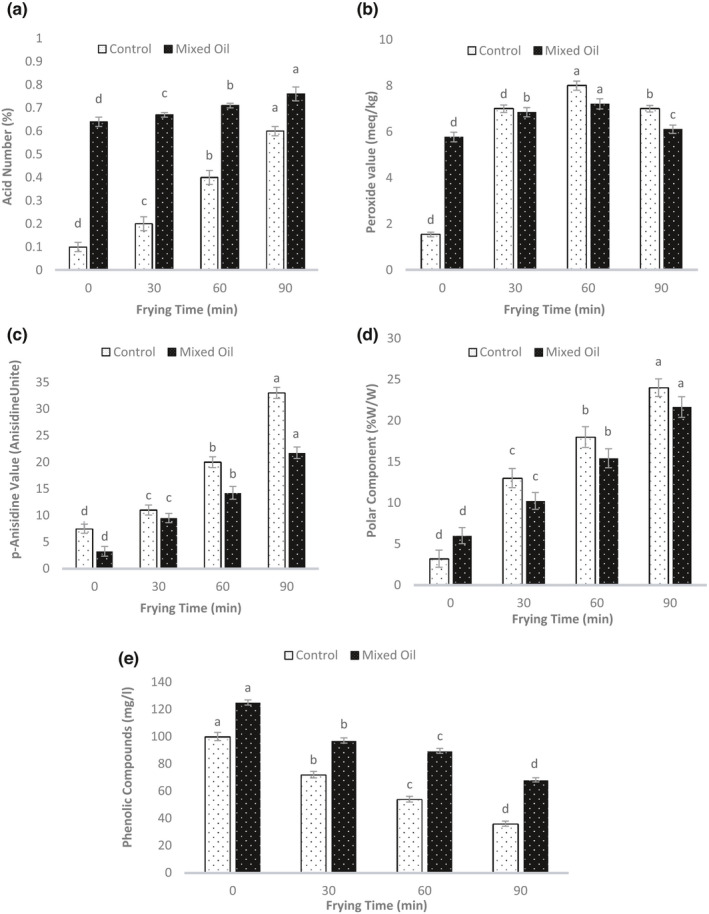
(a‐e) Changes in acid value (%), peroxide value (meq kg^−1^), p‐Anisidine value (Anisidine unit), polar compounds, and the percentage of phenolic compounds of commercial oil (control) and the oil optimum formulation (mixed oil) during frying process at 180°C, respectively. Different superscripts represent significant differences at *p* < .05

The changes in the acid value in commercial oil (control) and the optimal mixture of sesame oil and grapeseed oil during 90 min of frying at 180°C are presented in Figure 2a . According to this figure, by increasing the frying time the oil acid value increased significantly (*p* ≤ .05); the acid value of commercial oil was lower than the oil optimum formulation. The increase in the acid value during the high‐temperature heating process is attributed to the role of high temperature in accelerating fatty acid separation from oil molecules or facilitating the mixing of the fried food water with frying oil under this condition. In addition, the free fatty acids are capable of emulsifying water and oil, enhancing the hydrolysis, and ultimately increasing the number of free fatty acids. During the 90 min frying process, the acid value of oil optimum formulation and commercial oil increased to 0.7% and 0.6%, respectively. The trend of increasing free fatty acids in the oil with prolonged process time is in line with the findings of many researchers. Casal et al. (2010) reported during heating of five types of olive oil at high frying temperatures, fatty acids are hydrolyzed and released, which in turn increases the acid value and this action continues as the frying time increases. Kaviani et al. (2013) evaluated the spoilage time of canola oil based on the oxidative indicators during deep‐frying of potatoes. These researchers found that increasing the temperature of frying process led to an increase in the acid value. In addition, increasing of frying time at higher temperatures caused a more severe increase in the acid value. According to Codex Standard for Edible Fats and Oils (Alimentarius, 1999), the maximum acid value of virgin vegetable oils is 0.3% and 0.1% for the refined vegetable oils before heating. Also, according to INSO standard No. 4152 (INSO, 2014) the maximum acid value of frying vegetable oils is 0.2%. Therefore, the acid value of commercial oil used in this study was within the permissible limit of Iran National Standard (0.2%) for half an hour frying time but was higher than the limit for longer process times. In conclusion, the acid value of the optimum mixture of sesame oil and grapeseed oil (0.7%) is above the permissible limit of Iran's national standard.

#### 3.3.2 Peroxide value

Figure 2b shows the changes in peroxide value in the commercial oil (control) and the oil optimum formulation during 90 min of frying at 180°C. As shown in this figure, the peroxide value of oil samples increased steadily up to 60 min (from beginning of frying) then decreased until the end of the process (*p* ≤ .05). Since the peroxide value represents the primary oxidation products, the amount of peroxide increased in the early stage of frying. Then the peroxide value decreased when the unstable primary oxidation products transform into secondary products. The hydroperoxide decomposition means production of free radicals, thereby accelerating the oxidation reaction. Therefore, hydroperoxides have a high latent potential for intensifying oxidation activity. During frying process, the peroxide value of commercial oil was higher than the sesame and grapeseed oil optimum formulation. According to the Iranian National Standard No. 4152, the maximum permitted peroxide level for vegetable frying oils is 5 meq kg^−1^ oil. Based on the results shown in Figure 2b , the peroxide value of the commercial oil sample and the oil optimum formulation was not within the permissible limit of the National Iranian Standard. On the other hand, comparing the peroxide value of oil samples indicates that the peroxide value of commercial oil and the optimum formulation of sesame and grapeseed oil is within the acceptable limits of Codex standard (10 meq kg^−1^ oil) at the end of the heating process.

#### 3.3.3 p‐Anisidine value

The p‐Anisidine value is an indicator of the primary oxidation products breakdown level to the secondary oxidation products. In particular, it indicates the level of low volatility aldehydes. Therefore, this index is considered a sign of oxidation progress. The results of measuring the para Anisidine value of commercial oil and the oil optimum formulation during 90 min of frying at 180°C are shown in Figure 2c. During frying process, para Anisidine value of both commercial oil and oil optimum formulation increased significantly (*p* ≤ .05). However, this increase has a slightly steeper slope from the 60th min of the process. Interestingly, the 60th min of the process is the time of decreasing peroxide value (Figure 2b).

#### 3.3.4 Total polar compounds

Figure 2d shows the changes in polar compounds contents of oil samples during 90 min of frying at 180°C. As shown in Figure 2d, the percentage of polar compounds in both oil samples increased steadily with increasing of frying time (*p* ≤ .05). Polar compounds are derived from oxidation, polymerization, and hydrolysis of fat molecules. Free fatty acids, monoacylglycerols, diacylglycerols, aldehydes, ketones, dimers, and trimers are examples of polar compounds. Therefore, polar compounds are present even in newly extracted oils. Most of the world’s countries have a consensus on the reliable index of polar compounds as a measure of oil degradation during frying process. The maximum permissible amount of polar compounds, set by many national and international standards, is 24%–25% (w/w) which means the discarding time of frying oil (Bansal et al., 2010). In this study, the amount of polar compounds after 90 min of frying at 180°C reached 21.65% (w/w) in oil optimum formulation and 24% (w/w) in commercial oil that both values are within the permissible range.

#### 3.3.5 Total phenolic compounds

Phenolic compounds are antioxidants that prevent the onset of the oxidation process or slow down its rate by reacting with free lipid radicals. These compounds also react with the proxy and alkoxy radicals produced in the propagation step of the oxidation process. In this way, they also prevent the progression of oxidation (Hassanien & Abdel‐Razek, 2012). As a result, the presence of these compounds in the oil is profitable. The changes in the oil phenolic compounds by increasing the frying time are presented in Figure 2e. As shown in Figure 2e, the percentage of phenolic compounds significantly decreased (*p* < .05) by increasing the frying time and reached the lowest level at the end of the frying process (90 min). Polyphenols can be oxidized during frying and therefore decrease by increasing the frying time. Phenolic compounds of commercial oil and oil optimum formulation decreased to 36 and 68 mg l^−1^, respectively, after 90 min of frying.

## 4 CONCLUSION

In this study, three mixtures of sesame oil and grapeseed oil were prepared in different ratios and evaluated for physicochemical properties to increase the oil nutritional value and achieve a formulation with the best oxidative resistance. By increasing the sesame oil in the formulation, the iodine and peroxide values decreased while the phenolic compounds and antioxidant activity increased resulting in oxidative stability of formulated samples. The mixture of 75% sesame oil and 25% grapeseed oil had the best nutritional quality and the lowest price compared to the other oil formulas, so this formulation was selected as the optimum formulation. The effect of heating on different properties of the optimum formulation was investigated, too. The acid value of the optimum formulation increased significantly by increasing the frying time. By increasing the frying time, the peroxide value increased significantly but then decreased. The p‐Anisidine value also increased notably by the decomposition of peroxides. Phenolic compounds decreased by increasing the frying time. Overall, the optimum mixture of sesame oil and grapeseed oil is suitable to use as a cooking or salad oil.

## CONFLICT OF INTEREST

It is declared that there is no conflict of interest in the publication of this work.

## Author Contribution

**Maryam Khakbaz Heshmati:** Conceptualization (lead); Data curation (lead); Formal analysis (lead); Funding acquisition (lead); Investigation (lead); Methodology (lead); Project administration (lead); Resources (lead); Software (lead); Supervision (lead); Validation (lead); Visualization (lead); Writing – original draft (lead); Writing – review & editing (lead). **Maryam Jafarzadeh‐moghaddam:** Formal analysis (supporting); Investigation (supporting); Methodology (supporting); Software (supporting); Visualization (equal); Writing – original draft (equal). **akram pezeshki:** Methodology (supporting); Project administration (equal); Supervision (supporting); Validation (equal); Visualization (equal); Writing – original draft (supporting). **Rezvan Shaddel:** Data curation (equal); Methodology (supporting); Validation (equal); Visualization (equal); Writing – original draft (supporting); Writing – review & editing (lead).

## ETHICAL APPROVAL

Our research did not contain any animal experiments or human subjects.

## Data Availability

Data available on request from the authors.
